# Urinalysis, but Not Blood Biochemistry, Detects the Early Renal Impairment in Patients with COVID-19

**DOI:** 10.3390/diagnostics12030602

**Published:** 2022-02-27

**Authors:** Haifeng Zhou, Zili Zhang, Maria Dobrinina, Yalan Dong, Zhenyu Kang, Valerii Chereshnev, Desheng Hu, Zhe Zhang, Jun Zhang, Alexey Sarapultsev

**Affiliations:** 1 Department of Integrated Traditional Chinese and Western Medicine, Union Hospital, Tongji Medical College, Huazhong University of Science and Technology, Wuhan 430022, China; h_zhou@iip.uran.ru (H.Z.); zi_zhang@iip.uran.ru (Z.Z.); y_dong@iip.uran.ru (Y.D.); z_kang@iip.uran.ru (Z.K.); desheng.hu@hust.edu.cn (D.H.); 2 Institute of Immunology and Physiology, Ural Branch of the Russian Academy of Science, 620049 Ekaterinburg, Russia; mdobrynina87@gmail.com (M.D.); v.chereshnev@iip.uran.ru (V.C.); 3 Department of Physical Examination, The Central Hospital of Wuhan, Tongji Medical College, Huazhong University of Science and Technology, Wuhan 430014, China; z_zhang@iip.uran.ru; 4 Department of Critical Care Medicine, Wuhan Traditional Chinese Medicine Hospital, Wuhan 430014, China; 5 Russian—Chinese Education and Research Center of System Pathology, South Ural State University, 454080 Chelyabinsk, Russia

**Keywords:** acute kidney injury, AKI, COVID-19, disease severity, kidney impairment, urinalysis

## Abstract

Background: Coronavirus 2019 (COVID-19), caused by the severe acute respiratory syndrome coronavirus (SARS-CoV-2), has created a tremendous economic and medical burden. The prevalence and prognostic value of SARS-CoV-2-induced kidney impairment remain controversial. The current study aimed to provide additional evidence on the incidence of acute kidney injury (AKI) in COVID-19 patients and propose the use of urinalysis as a tool for screening kidney impairment. Methods: 178 patients with confirmed COVID-19 were enrolled in this retrospective cohort study. The laboratory examinations included routine blood tests, blood biochemical analyses (liver function, renal function, lipids, and glucose), blood coagulation index, lymphocyte subset and cytokine analysis, urine routine test, C-reactive protein, erythrocyte sedimentation, and serum ferritin. Results: No patient exhibited a rise in serum creatinine or Cystatin C and occurrence of AKI, and only 2.8% of patients were recorded with an elevated level of blood urea nitrogen among all cases. On the contrary, 54.2% of patients who underwent routine urine testing presented with an abnormal urinalysis as featured by proteinuria, hematuria, and leucocyturia. Conclusions: Kidney impairment is prevalent among COVID-19 patients, with an abnormal urinalysis as a clinical manifestation, implying that a routine urine test is a stronger indication of prospective kidney complication than a blood biochemistry test.

## 1. Introduction

Since its outbreak in 2019, Coronavirus 2019 (COVID-19), caused by the new severe acute respiratory syndrome coronavirus (SARS-CoV-2), has quickly swept over the globe, resulting in a global pandemic. There have been over 279 million confirmed cases of COVID-19 as of 27 December 2021, with 5.3 million deaths reported to the WHO, placing a tremendous economic and medical burden on the world.

SARS-CoV-2 uses angiotensin-converting enzyme II (ACE2) as a cell entry receptor; thus, patients with COVID-19 are likely to develop multiple organ or tissue injuries [1,2,3]. It is reported that patients with SARS-CoV-2 infection show elevated levels of alanine aminotransferase (ALT), aspartate aminotransferase (AST), and gamma-glutamyl transferase (GGT), indicating liver and myocardial damage [4]. The probability of major complications and death in COVID-19 patients increases with age, especially in persons with metabolic syndrome, type 2 diabetes mellitus, and some other serious chronic diseases, which are considered to be risk factors for the severe course of COVID-19 [5,6,7,8]. COVID-19, in turn, is a complicating factor for other critical diseases, such as burn injury and surgical complications [9].

ARDS is the most common critical complication of COVID-19 pneumonia (approximately 20%) and one of the leading causes of death [10,11,12,13]. For its part, ARDS leads to the development of critical hypoxia and systemic inflammation and, as a clinical reflection of systemic inflammation, to a variety of resuscitation syndromes, including multiple organ dysfunction syndrome (MODS) and DIC syndrome [14].

Therefore, the prevalence and prognostic value of SARS-CoV-2-induced kidney impairment or dysfunction remain controversial issues. The first studies, which mainly were conducted on patients in China, recorded acute kidney injury (AKI) prevalence in the range of 3.6–7 [5,15,16]. Moreover, the study of Guan WJ et al. (2020) revealed only 0.5% of patients developed AKI, while Wang et al. (2020) reported that SARS-CoV-2 infection could neither cause obvious AKI nor aggravate CRF in the COVID-19 patients [17,18]. In contrast to those findings, however, a single-centered observational study by Yang X (2020) found that 29% of patients with severe SARS-CoV-2 pneumonia developed acute kidney injury (AKI) [19]. Similar results were obtained in the retrospective cohort studies of J.S Bell (2021) and Li Z (2020) and described in the systematic reviews of SA Silver (2021) and R Raina [20,21,22,23]. According to those studies, AKI occurred in over a quarter of patients hospitalized with COVID-19 [20,21,22,23].

As a matter of fact, the incidence of AKI varies across geographic locations, reaching 28.6% among hospitalized COVID-19 patients in the USA and Europe, while being only about 5.5% among patients in China [24]. Moreover, AKI is associated with significantly increased mortality in COVID-19 patients with higher AKI stage, portending a worse prognosis [20]. As a result, it is critical to develop sensitive evaluation markers or models for identifying patients at high risk of exacerbation who could benefit from early management to prevent complications and mortality.

The current study aimed to provide additional evidence on the incidence of kidney injury in COVID-19 patients and propose the use of urinalysis as an effective tool for screening kidney impairment and predicting disease severity.

## 2. Materials and Methods

### 2.1. Study Design

**Figure 1:**
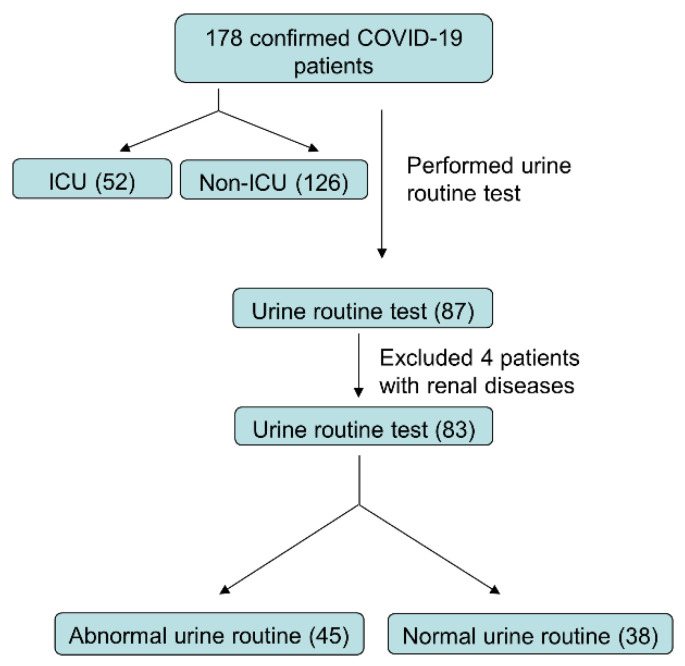
**The flow chart of COVID-19 patients inclusion procedure**.

From 2 February to 29 February 2020, 178 patients with confirmed COVID-19 were enrolled in this retrospective study at Wuhan Union Hospital, Tongji Medical College, Huazhong University of Science and Technology (Wuhan, China). All patients in this study were diagnosed according to the guidance (fifth edition) published by the Chinese National Health Commission. Patients with a history of kidney disease were excluded from the study (Figure 1). 

The study was approved by the Institutional Ethics Board of Wuhan Union Hospital of Tongji Medical College, Huazhong University of Science and Technology (No. Union Hospital-0093). Written informed consent was waived by the Ethics Commission of the designated hospital for emerging infectious diseases.

### 2.2. Data Collection

The demographic data, symptoms and vital signs, and the results of laboratory examinations were retrieved from electronic medical records for this study. The laboratory examinations included blood routine test, biochemical blood tests (liver function, renal function, lipids, and glucose), blood coagulation index, lymphocyte subset and cytokine analysis, urine routine test, C-reactive protein (CRP), erythrocyte sedimentation (ESR), serum ferritin (SF), etc. The Chronic Kidney Disease Epidemiology Collaboration (CKD-EPI) equation was used to calculate the estimated glomerular filtration rate (eGFR) [25]. Patients were assessed daily for the presence of AKI or renal failure using strict criteria based on the KDIGO 2012 Clinical Practice Guideline for AKI [26]. The severity of the disease was determined as described in a previous study [27], according to the diagnostic and treatment guideline for SARS-CoV-2 issued by the Chinese National Health Committee.

All laboratory tests were performed at Union Hospital’s clinical laboratory department, and the normal range of these tests was provided by them. The threshold values were as follows: serum creatinine (Scr) 54–133 (μmol/L) for males and 44–106 (μmol/L) for females, blood urea nitrogen (BUN) 2.9–8.2 (mmol/L), serum uric acid 208–428 (μmol/L) for males and 155–357 (μmol/L) for females, and Cystatin C 0.63–1.25 (mg/L) for males and 0.54–1.15 (mg/L) for females. The urine samples were collected in sterile containers and processed within 2 h of collection. Urinalysis measurements were performed by automated urine analyzer UC-3500 for chemical analysis and UF-1000i for sediment analysis. In addition, the reliability of measurements was further assured by manual microscopic analysis, if required. Urine sediments were collected by centrifugation for 15 min at 500× *g*, and a drop of pellets was placed on a glass slide, covered with a cover slip, and immediately examined using a light microscope. Proteinuria was defined as positive in urine protein (semiquantitative, +−/+/++/+++), and hematuria was recorded with elevated urine erythrocyte quantification (>17/μL) as well as positive in semiquantitative urinalysis (+−/+/++/+++). Due to lack of data, all of the parameters were examined using extreme values, with the exception of urinalysis at the first examination after admission.

### 2.3. Statistical Analysis

The categorical variables were summarized as counts or percentages, and the continuous variables were expressed as mean ± SD or medians and interquartile ranges (IQR) as appropriate. Categorical variables were compared using the chi-square test; the Fisher exact test was used where data were limited. Where data were normally distributed, an independent group t-test was employed to compare continuous variables; otherwise, the Mann–Whitney test was used. All statistical analyses were performed using SPSS 20.0 software (SPSS Inc., Chicago, IL, USA), and *p* < 0.05 was regarded as a significant difference.

## 3. Results

### 3.1. Presenting Characteristics

A total of 178 patients with laboratory-confirmed SARS-CoV-2 infection were enrolled in this study, among which 52 patients were from ICU, and 126 patients were from ordinary isolation wards. As shown in Table 1, the median age of the patients in the ICU group was 56.5 years (IQR, 40.50–65.5), which was significantly higher than that of the patients in the non-ICU group (45, IQR [32.75–57.00]). For all the patients, the most common symptoms were fever (83.1%) and cough (71.9%). Compared with Non-ICU patients, the ICU patients showed no difference in fever, shortness of breath, chest tightness, fatigue, myalgia, and headache but were more likely to experience cough, hypoxemia, oxygen application. 

The proportion of males in the ICU group was higher than that in the non-ICU group (61.5% vs. 31.7%). More patients in the ICU group compared to the non-ICU group had a high body temperature (T > 38 °C) (75% vs. 56.3%). Additionally, some underlying diseases were more common in ICU patients, such as hypertension (28.8% vs. 11.1%), diabetes (17.3% vs. 7.9%), cardiovascular insults (13.5% vs. 2.4%), digestive system (7.7% vs. 4.8%), and respiratory system disease (7.7% vs. 4.8%). These data were consistent with recent reports related to COVID-19 that older men with comorbidities are more likely to be infected and progress into severe cases and even die due to weaker immune function.

### 3.2. No COVID-19 Patients Diagnosed with AKI

It was discovered in previous studies that many patients with SARS-CoV-2 infection usually had increased levels of Scr, BUN, and even AKI, especially those with severe conditions [15,16,17,18,19,20,21,22,23]. These clinical signs, however, were unexpectedly absent from our findings. Patients in both the ICU group and non-ICU group displayed normal Scr and cystatin C levels. Only 2.8 percent of the patients had an elevated BUN level, despite the fact that 23.6 percent of the patients had a lower estimated glomerular filtration rate (eGFR) against the usual range. In line with these findings, none of the patients had AKI throughout their hospital stay, regardless of being admitted to the ICU or not. In summary, our findings showed that AKI was not present in our COVID-19 patients.

### 3.3. Potential Kidney Impairment Reflected by the Routine Urine Test

Urine examination results were taken to further analyze the kidney impairment in detail, considering the fact that Scr and BUN may not be changed due to the high compensatory adaptability of the kidney. A regular urine routine test was not administered to each and every COVID-19 patient. Only 87 of the 178 patients involved in the study had their urine tested upon admission, and four of them had a history of kidney disease, including diabetic nephropathy, kidney stones, multiple renal cystic diseases, and kidney carcinoma. Those four patients were also left out of our subsequent study. The remaining 83 patients were selected, as illustrated in Figure 1, with a participation rate of 46.6%. None of the 83 patients had any similar symptoms or was diagnosed with a urinary tract infection, urethral injury, or other diseases. Surprisingly, 54.2 percent of the patients (45/83) had abnormal urine test results, such as proteinuria, hematuria, and leukocyturia (Table 2).

**Figure 2:**
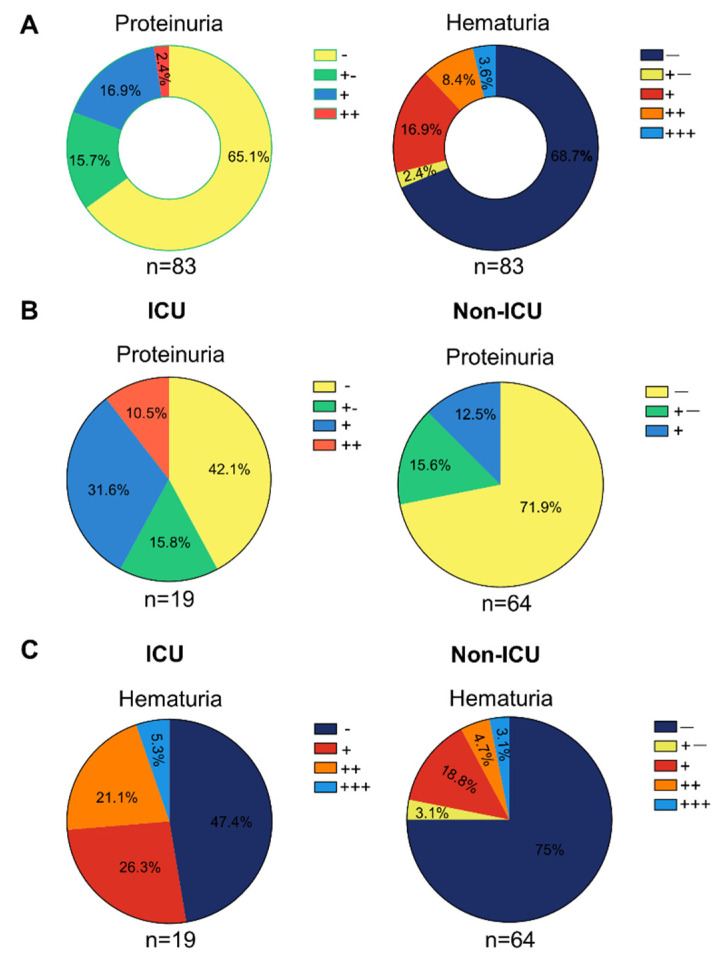
**Pie chart illustrating COVID-19 patients exhibited abnormal urinalysis, including proteinuria and hematuria.** (**A**) Analysis of all the COVID-19 patients with urine routine test. (**B**) Analysis of proteinuria between ICU and non-ICU. (**C**) Analysis of hematuria between ICU and non-ICU.

Specifically, 34.9% of the cases had positive urine protein, with 15.7% for “+−”, 16.9% for “+”, and 2.4% for “++” (Figure 2A). At the same time, 31.3% of the patients presented with hematuria, including 2.4% for “+−”, 16.9% for “+”, 8.4% for “++”, and 3.6% for “+++” (Figure 2A). In addition, the patients in ICU exhibited more severe urinalysis results com-pared with non-ICU patients, such as proteinuria (+, 31.6% vs. 12.5%; ++, 10.5% vs. 0) and hematuria (+, 26.3% vs. 18.8%; ++, 21.1% vs. 4.7%; +++, 5.3% vs. 3.1%) (*p* < 0.05 for all) (Figure 2B,C). Additionally, consistent with a previous report [28], the specific urine weight is higher in ICU patients compared to non-ICU patients (Table 2). These results indicated the prevalence of kidney impairment among COVID-19 patients.

### 3.4. Kidney Impairment Was Caused by a Direct Virus Invading

Given that proteinuria and hematuria were two prominent signs of kidney impairment, 83 patients were allocated to groups of abnormal urinalysis (AU) or normal urinalysis (NU) by test results. It is also possible that the kidney impairment was due to drug nephrotoxicity. The medications of the patients before admission included antibiotics (Moxifloxacin, Azithromycin, Amoxicillin) (30.1%), antiviral drugs (Oseltamivir, Lopinavir, Arbidol) (33.8%), and Chinese patent medicine (Lianhuaqingwen capsule, Isatis root granule) (10.8%). However, there was no statistical difference in prehospital treatment between the AU and NU groups (Table 3), suggesting that the kidney impairment was caused by SARS-CoV-2 infection rather than treatment agents.

### 3.5. Urinalysis Abnormality Correlates with the Severity of COVID-19

**Figure 3:**
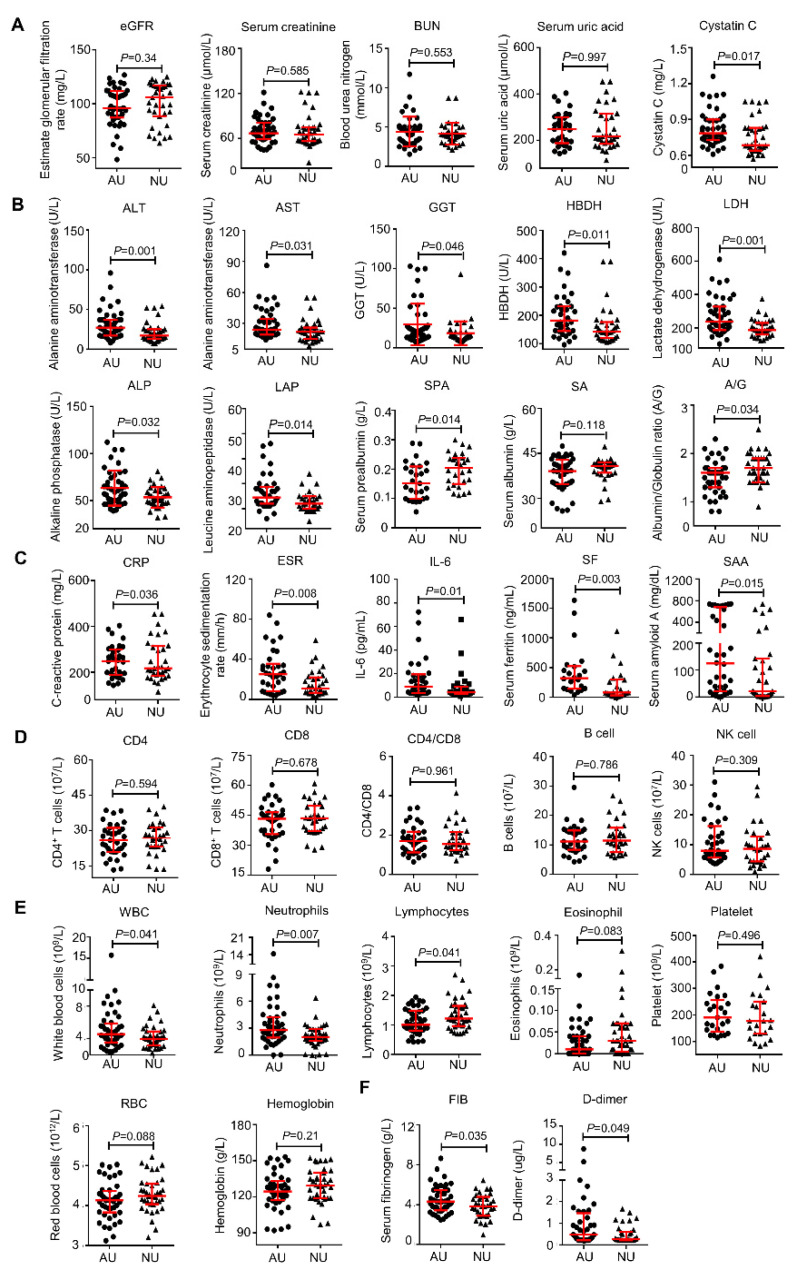
**The comparison of laboratory parameters between abnormal urinalysis group and normal urinalysis group.** Analysis of renal function (**A**), analysis of liver function (**B**), inflammation (**C**), lymphocytes subpopulations (**D**), blood routine (**E**), and coagulation function (**F**). Abbreviation: GGT, γ-glutamyl transpeptidase; HBDH: α-hydroxybutyrate dehydrogenase.

Subsequently, to explore whether urinalysis correlates with disease severity, we compared the differences in other laboratory parameters between the AU and NU groups. Firstly, we found that there was no difference in eGFR, Scr, BUN, and serum uric acid between the two groups. Although the patients with AU had a higher level of cystatin C, all the values were generally within the normal range (Figure 3A). Next, as shown in Figure 3B, the patients in the AU group demonstrated a markedly higher level of liver injury related indicators, such as ALT, AST, GGT, α-hydroxybutyrate dehydrogenase (HBDH), lactic dehydrogenase (LDH), alkaline phosphatase (ALP), and leucine aminopeptidase (LAP). Moreover, both serum albumin level and albumin-globulin ratio (A/G), markers related to synthetic liver capability and inflammatory state were lower in the AU group than in the NU group, indicating possible liver inflammation. These data suggest that patients with an abnormal urinalysis might be susceptible to liver damage. In addition, the patients in the AU group usually presented with higher levels of inflammation-related markers, including C-reactive protein (CRP), erythrocyte sedimentation rate (ESR), IL-6, serum ferritin (SF), and serum amyloid A (SAA) (Figure 3C). However, no significant differences were observed in major lymphocytes subpopulations, such as CD4+ T cells, CD8+ T cells, CD4/CD8, B cells, and NK cells (Figure 3D), whereas the absolute count of peripheral lymphocytes in AU patients was significantly lower than that of NU patients, and white blood cell (WBC) and neutrophil counts were usually higher in patients in the AU group (Figure 3E). There was no significant difference between the two groups in platelet, red blood cells (RBC), and hemoglobin (Figure 3E). As for the coagulation profile, the AU patients had statistically higher levels of serum fibrinogen (FIB) and D-dimer than NU patients, indicating a worse coagulation function (Figure 3F). Collectively, these results indicated that urinalysis abnormality correlates with the severity of COVID-19, and a urine routine test may be a good method for predicting the disease progression in COVID-19 patients.

## 4. Discussion

Our findings imply that kidney damage is common in SARS-CoV-2 infection patients, as evidenced by abnormal urinalyses, such as proteinuria, hematuria, and leukocyturia. AKI, on the other hand, was not present in our group. 

Previous papers demonstrated renal impairment in COVID-19 patients [19,20,21,22,23]. However, the proportion of increased Scr or incidence of AKI after SARS-CoV-2 infection was quite different among these studies. This may be attributable to many factors, including the time point of testing and different proportion of severe cases in studied countries, and/or different scales of patient sampling. Furthermore, several publications reported the important role of urine tests in evaluating the severity, occurrence of AKI, and clinical course of COVID-19 patients, supporting the utility of urine as an informative biospecimen [29,30,31]. 

In the current study, we enrolled 178 patients with confirmed SARS-CoV-2 infection, including 52 cases admitted to ICU and 126 cases not admitted to ICU. Regarding kidney function indicators from blood biochemistry, we found no patient exhibited a rise in Scr or Cystatin C and no occurrence of AKI both in either ICU group and or non-ICU group, and only 2.8% of the patients were recorded with an elevated level of BUN among all cases. On the contrary, 54.2% (45/83) of the patients who had performed urine routine tests (48.9% [87/178]) presented with an abnormal urinalysis, which was as featured by proteinuria, hematuria, and leucocyturia. These findings show that renal impairment is widespread in both severe and non-severe COVID-19 individuals and that abnormal urine routine tests are more sensitive than blood biochemistry markers such as high Scr and BUN. These results intersect with those of O Gross et al. (2021), who also recognized the relevance of urinalysis and designated it as the first inspector in a proposed COVID-19 risk assessment algorithm [32].

The stark differences between blood chemistry analysis and urinalysis piqued our interest, so we looked into the explanations. To begin with, even though SARS-CoV-2 infection is known to cause renal impairment, clinical symptoms were not obvious due to the kidney’s powerful compensatory function. It is known that the serum levels of Scr and BUN will exceed the normal range only when more than 50% of kidney function has been lost. Thus, the severity and/or duration of COVID-19 maybe not be enough to result in elevated plasma markers related to renal function. 

**Figure 4:**
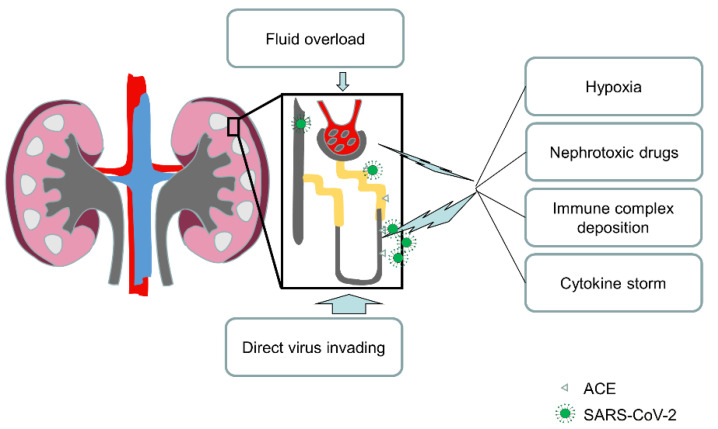
**Potential mechanisms of kidney impairment in COVID-19 patients.** SARS-CoV-2 can directly invade renal tubular epithelial cells via ACE2, a virus-specific receptor that is mainly expressed in proximal tubular cells, which potentially leads to host cell damage and death. At the same time, nephrotoxic drugs, hypoxia, and infectious induced extensive immune activation featured by cytokine storm and immune complex formation were also factors related to tubular cell impairment. The impairment caused by compromise of tubular integrity may be major reason for the occurrence of an abnormal urinalysis. In addition, although there were no significant signs of kidney dysfunction and acute kidney injury characterized by increased Scr, BUN, and cystatin C, many known factors may provoke injury to the glomeruli, such as inflammation, nephrotoxic drugs, and fluid overload.

Secondly, the kidney damage in COVID-19 patients may be confined in renal tubules rather than glomerulus (Figure 4) [33,34,35]. 

SARS-CoV-2 enters host cells via connecting to the ACE2 receptor on the cell surface. At the same time, ACE2 immunostaining studies demonstrated that the mesangium and glomerular endothelium were ACE2 negative, and the distal tubules and collecting ducts had weak cytoplasmic staining whereas the brush border of proximal tubular cells had extensive staining [36]. Moreover, two studies conducted by Xu et al. and Qi’s group demonstrated that proximal straight tubule cells were potential host cells targeted by SARS-CoV-2 by scRNA-seq analysis [37,38].

B Diao et al. (2021) later confirmed this assumption by revealing that SARS-CoV-2 NP expression was present in tubules and was missing in the glomerulus of kidneys from COVID-19 autopsies [39]. These were found to be focally hemorrhagic, exhibiting varying degrees of acute tubular necrosis, but no glomerular pathology or cellular infiltrates were discovered [39].

Furthermore, SARS-CoV was found in epithelial cells of distal convoluted renal tubules in another study on the organ distribution of SARS-CoV in people who died of SARS [40]. Meanwhile, in a pathological examination of kidney tissues from seven SARS patient autopsies, no glomerular pathology was revealed in the kidneys, but all seven renal specimens displayed acute tubular necrosis of various degrees [41]. Finally, Su et al. presented direct evidence of SARS-CoV-2 invasion into the kidney and discovered remarkably extensive proximal tubule damage in the autopsies of COVID-19 patients [42].

Gender, age, underlying disorders, and laboratory tests are just a few of the characteristics and models that have so far been constructed to predict changes in condition among COVID-19 patients [13,27,43,44,45,46]. In order to evaluate whether urinalysis is useful for predicting the disease severity, we divided the patients into two groups based on their results of urine routine test on admission, designated as abnormal urinalysis group (AU) and normal urinalysis group (NU). Compared to the NU group, patients in the AU group presented no difference in BUN, Scr, and eGFR but had elevated cystatin C. In addition, we found that patients with an abnormal urinalysis usually had more severe laboratory parameters, such as higher liver and heart injury index, including ALT, AST, ALP, LDH, HBDH, GGT, and LAP; inflammation-related markers, including CRP, IL-6, ESR, SAA, and serum ferritin; blood routine indicator including WBC, neutrophils, and lymphocytes; and related coagulation indicator including FIB and D-dimer. This analysis revealed that the patients with kidney impairment were generally in worse condition, including multiple organ or tissue injuries (liver, heart), inflammation, and hypercoagulability state, which was probably due to low immunity and high viral load. In addition, our study indicates that urinalysis, an inexpensive, fast, and easy test to perform, is an excellent method to predict a severe condition in COVID-19 patients.

Admittedly, several issues need to be addressed in this study. Firstly, no dynamic analysis of urinalysis was not performed for the COVID-19 patients due to the fact that many of the patients only had one urine routine test on admission. Secondly, a larger sample size should be enrolled in our study. In addition, the protein measurements in urine by additional biochemical method to verify the urinalysis results is necessary. We will attempt to address these questions in our future research.

## 5. Conclusions

We demonstrated that kidney impairment is prevalent among COVID-19 patients, with an abnormal urinalysis as a clinical manifestation, implying that a urine routine test is a stronger indication of prospective kidney impairment than a blood chemistry test. Furthermore, our findings have shown that urinalysis might be used to predict the severity of the disease. As a result, we encourage front-line healthcare workers to pay more attention to renal impairment in SARS-CoV-2 infected patients and to monitor urinalysis to identify possible kidney impairment routinely.

## Figures and Tables

**Table 1:** Basic information and blood indicators related to kidney injury.

	Total (*n* = 178)	ICU (*n* = 52)	Non-ICU (*n* = 126)	^a^*p* Value
Age (Years)	47.0 (35.0–61.0)	56.50 (40.5–65.5)	45.0 (32.8–57.0)	<0.001
Gender				
male	72 (40.4)	32 (61.5)	40 (31.7)	<0.001
female	106 (59.6)	20 (38.5)	86 (68.3)	<0.001
**Signs and symptoms**				
Fever	148 (83.1)	46 (88.5)	100 (79.4)	0.199
Body temperature (max.) (°C)	38.5 (37.8–39.0)	39 (38.1–39.1)	38.3 (37.7–39.0)	0.005
>38	110 (61.8)	39 (75)	71 (56.3)	0.020
Shortness of breath	39 (21.9)	13 (25)	26 (20.6)	0.553
Cough	128 (71.9)	49 (94.2)	79 (62.3)	<0.001
Hypoxemia	30 (16.9)	17 (32.7)	13 (10.3)	<0.001
Chest tightness	35 (19.7)	13 (25)	22 (17.5)	0.300
Fatigue	41 (23.0)	16 (30.8)	25 (19.8)	0.122
Myalgia	49 (27.5)	17 (32.7)	32 (25.4)	0.358
Headache	12 (6.7)	4 (7.7)	8 (6.3)	0.748
Oxygen application	121 (68.0)	50 (96.2)	71 (56.3)	<0.001
Severe case	81 (45.5)	45 (86.5)	36 (28.6)	<0.001
**Basic diseases**				
hypertension	29 (16.3)	15 (28.8)	14 (11.1)	0.007
hyperlipidemia	3 (1.7)	1 (1.9)	2 (1.6)	>0.999
diabetes	19 (10.7)	9 (17.3)	10 (7.9)	0.106
digestive system	10 (5.6)	4 (7.7)	6 (4.8)	0.481
respiratory system	10 (5.6)	4 (7.7)	6 (4.8)	0.481
cardiovascular	10 (5.6)	7(13.5)	3 (2.4)	0.007
cancer	8 (4.5)	1 (1.9)	7 (5.6)	0.440
urinary system	4 (2.2)	2 (3.8)	2 (1.6)	0.581
Others	23 (12.9)	5 (9.6)	18 (14.3)	0.47
Non-basic diseases	100 (56.2)	19 (36.5)	81 (64.3)	<0.001
Scr (μmol/L)	65.2 (56.8–74.8)	71.0 (55.8–89.4)	65.3 (56.5–74.3)	0.067
increase	0	0	0	>0.999
BUN (mmol/L)	3.81 (2.83–4.54)	4.14 (3.33–4.82)	3.72 (2.76–4.57)	0.078
increase	5 (2.8)	3 (5.8)	2 (1.6)	0.150
eGFR (ml/min)	214.6 (308.05)	93.7 (78.1–108.0)	102.5 (90.7–113.1)	0.014
decrease	42 (23.6)	19 (36.5)	23 (18.3)	0.009
Cystatin C (mg/L)	0.77 (0.68–0.86)	0.81 (0.73–0.88)	0.77 (0.68–0.87)	0.155
increase	3 (1.7)	1 (1.9)	2 (1.6)	>0.999

Note: Data are shown as median (IQR) or *n* (%) as appropriate. ICU = intensive care unit, Scr = serum creatinine, BUN = blood urea nitrogen, eGFR = estimated glomerular filtration rate, IQR = interquartile range. ^a^ *p* values indicate differences between ICU and Non-ICU patients. *p* < 0.05 was considered statistically significant.

**Table 2:** Abnormal urinalysis results in COVID-19.

	Total (*n* = 83)	ICU (*n* = 19)	Non-IC (*n* = 64)	^a^*p* Value
Abnormal urine routine	45 (54.2)	15 (78.9)	30 (46.9)	0.014
Proteinuria				
positive	29 (34.9)	11(57.9)	18 (28.1)	0.017
Hematuria				
positive	24 (28.9)	10 (52.6)	14 (21.9)	0.009
Leucocyturia				
positive	14 (16.9)	2 (10.5)	12 (18.8)	0.506
Urine glucose				
positive	0 (0)	0 (0)	0 (0)	>0.999
Urine urothelial cell				
positive	10 (12)	1 (5.3)	9 (14.1)	0.441
Urine specific weight	1.022 ± 0.00118	1.027 ± 0.011	1.021 ± 0.012	0.003

Note: Data are shown as *n* (%) or mean ± standard deviation as appropriate. COVID-19 = novel coronavirus disease 2019, ICU = intensive care unit. ^a^ *p* values indicate differences between ICU and Non-ICU patients. *p* < 0.05 was considered statistically significant.

**Table 3:** Prehospital medications between AU and NU groups.

Medications before Admission	Total (*n* = 83)	AU (*n* = 45)	NU (*n* = 38)	^a^*p* Value
Antibiotic	25 (30.1)	11 (35.5)	14 (26.9)	0.411
Oseltamivir/Lopinavir	12 (14.5)	7 (22.6)	5 (9.6)	0.119
Arbidol	16 (19.3)	9 (29.0)	7 (13.5)	0.082
Chinese Patent Medicine	9 (10.8)	3 (9.7)	6 (11.5)	>0.999

Data are shown as *n* (%). AU = abnormal urinalysis group, NU = normal urinalysis group. ^a^ *p* values indicate differences between AU and NU patients. Chinese Patent Medicine consists of *Lianhuaqingwen* capsule and *Isatis* root granule. *p* < 0.05 was considered statistically significant.

## Data Availability

The datasets analyzed during the current study are available from the corresponding author on reasonable request as they contain information on the gender, age, work experience, and places of work of the respondents.
